# The American Society for the Study of Alcohol and Other Narcotics

**Published:** 1904-09

**Authors:** T. D. Crothers

**Affiliations:** Hartford, Conn.


### CORRESPONDENCE.

### The American Society for the Study of Alcohol and Other Narcotics.

*Editor Buffalo Medical Journal:*

Sir—The American Medical Society for the Study of Alcohol and other Narcotics was organised June 8, 1904, by the union of the American Association for the Study of Inebriety and the Medical Temperance Association. Both of these societies are composed of physicians interested in the study and treatment of inebriety and the physiological nature and action of alcohol and narcotics in health and disease. The first society was organised in 1870 and has published five volumes of transactions and twenty-seven yearly volumes of the *Quarterly Journal of Inebriety*, the organ of its association. The second society began in 1891 and has issued three volumes of transactions, and for seven years published a quarterly bulletin containing the papers read at its meetings.

The special object of the union of the two societies is to create greater interest among physicians to study one of the greatest evils of modern times. Its plan of work is, first, to encourage and promote more exact scientific studies of the nature and effects of alcohol in health and disease, particularly of its etiological, physiological and therapeutic relations; second, to secure more accurate investigations of the diseases associated with or following the use of alcohol and narcotics; third, to correct the empirical treatment of these diseases by secret drugs and so-called specifics and to secure legislation, prohibiting the sale of nostrums claiming to be absolute cures containing dangerous poisons; and, fourth, to encourage special legislation for the care, control and medical treatment of spirit and drug-takers.

The alcoholic problem and the diseases which center and spring from it are becoming more prominent and its medical and hygienic importance has assumed such proportions that physicians everywhere are called on for advice and counsel. Public sentiment is turning to medical men for authoritative facts and conclusions to enable them to realise the causes, means of prevention and cure of this evil. This new society comes to meet this want by enlisting medical men as members and stimulating new studies and researches from a broader and more scientific point of view.

As a medical and hygienic topic the alcoholic problem has an intense personal interest, not only to every physician, but to the public generally in every town and city in the country. This interest demands concentrated efforts through the medium of a society to clear away the present confusion, educate public sentiment, and make medical men the final authority in the consideration of the remedial measures for cure and prevention. For this purpose a most urgent appeal is made to all physicians to assist in making this society the medium and authority for the scientific study of the subject. The undersigned will be pleased to give any further information.

T. D. Crothers, M. D., *Secretary,*
Hartford, Conn.

